# The Effects of High-Fidelity Simulation on Salivary Cortisol Levels in SRNA Students: A Pilot Study

**DOI:** 10.1100/tsw.2011.8

**Published:** 2011-01-05

**Authors:** Terri Jones, Sarah Goss, Bethany Weeks, Hiroko Miura, Damian Bassandeh, Dennis Cheek

**Affiliations:** ^1^ School of Nurse Anesthesia, Harris College of Nursing and Health Sciences, Texas Christian University, Fort Worth, TX, USA; ^2^ Nursing Research Lab, Harris College of Nursing and Health Sciences, Texas Christian University, Fort Worth, TX, USA

**Keywords:** salivary cortisol, high-fidelity simulation, anesthesia, SRNA students, health care, pilot study

## Abstract

The use of clinical simulation in graduate level nursing education provides the opportunity for students to learn and apply theoretical practices of nursing care in a safe and controlled environment. It was postulated that laboratory simulation would mimic the stress levels of a real clinical situation as measured by the stress hormone cortisol. The purpose of this study was to determine whether high-fidelity simulation approximates the stress experienced by nurse anesthesia students in the operating room. Participants (n = 21) were recruited from an accredited nurse anesthesia program in the southern U.S. Saliva was collected for 3 days under controlled conditions for baseline data. Next, saliva was collected for 3 days: the day before, the day of, and the day after simulation. The same process was repeated for the first clinical day in the operating room. The participants acted as their own control. There was a significant (*p* < 0.05) increase in cortisol levels during laboratory simulation as compared to baseline values. Although levels of cortisol were higher during clinical time than baseline, this increase was not significant (*p* > 0.05), and levels were lower than levels during simulation. Laboratory simulation of patient scenarios raised the stress hormone cortisol level threefold above baseline levels in nurse anesthesia students, while actual clinical experience did not.

## INTRODUCTION

Many nurse anesthesia programs incorporate operating room (OR) simulations into their health care curriculum. Patient simulation as a health education tool began in the 1950s, but its use is rising due to declining inpatient populations, concerns for patient safety, and advances in learning theory. Increasingly, health care educators look to high-fidelity patient simulators as an effective way for students to acquire clinical skills[1]. It is also an ideal setting in which to evaluate how these students react in an emergent situation. High-fidelity simulation correlates with increased nursing student satisfaction as well as self-confidence[2]. Previous studies of patient simulation and its use in clinical education and training have considered cortisol levels as an outcome measurement[3]. Scarce data exist on the amount of stress that participants feel during patient simulation exercises. Using cortisol as a biomarker for stress levels provides a scientific indicator of the amount of stress experienced during simulation training.

Cortisol is a hormone released from the adrenal cortex. The functions of cortisol include, but are not limited to, regulation of blood sugar, maintenance of blood pressure, and anti-inflammatory action in response to stress[4]. Cortisol levels rise due to circadian influences as well as numerous environmental changes, in particular, stress[5]. One can measure cortisol via serum or salivary samples. Collection of salivary cortisol has the advantages of being noninvasive, fast, and simple since participants can collect their samples independently, as opposed to blood draws, which require a needle stick by a trained professional. Research shows that circadian profiles for salivary cortisol closely parallel those of circulating serum cortisol concentrations over a 24-h period[6].

The purpose of this study was to evaluate the effect of high-fidelity simulation on salivary cortisol levels in student registered nurse anesthetists (SRNAs) since little research has been conducted in this area. In addition, cortisol levels from high-fidelity simulation were compared to cortisol levels in the OR in order to determine the ability of high-fidelity simulation to induce stress similar to that experienced in the OR. It was postulated that the simulation would induce stress, thus raising cortisol levels above participants' baseline levels, and would be similar to those seen in OR settings.

The National Council of State Board of Nursing (NCSBN) defines simulation as an educational process[7]. Both undergraduate and graduate nursing programs incorporate simulation as a part of the curriculum to enhance active learning. High-fidelity simulation creates the perception of reality and is an opportunity for application of cognitive, affective, and psychomotor learning[8]. Studies have demonstrated that high-fidelity simulation increases student satisfaction and confidence[2]. Many studies justify simulation as an effective tool to enhance learning; few studies use physiological evidence. By testing salivary cortisol levels, which reflect acute stress, a quantifiable measurement may be correlated to like situations[4].

High-fidelity simulation places learners in complex situations, allowing them to see cues and consequences that reflect reality[9]. Simulation is particularly conducive to learning if it involves instructor feedback and repetition of skills[9,10]. A correlation between time spent with high-fidelity simulators and standardized learning outcomes does exist[10,11]. Repetition of psychomotor, critical thinking, and communication skills in a brief period of time is an educational aspect of simulation that actual patient-care experiences cannot routinely offer. In addition, analysis of the student's performance, through a formal debriefing process, provides an opportunity for learning. In a study of 50 students by Gordon and Buckley, 94% of students agreed that a debriefing was the most valuable aspect of simulation[12].

While many studies reported positive outcomes in relation to simulation as an educational tool, few studies have evaluated the ability of simulation to induce stress. Girzadas et al. found that heart rate, self-reported stress, and learning were equally affected among participants in a critical airway scenario, regardless of the role played[13]. Another study performed in 2009 by Müllera et al. found that salivary cortisol levels increased during high-fidelity simulation[14]. Despite these data, no study has compared the ability of high-fidelity simulation to induce stress comparable to that of the actual OR using cortisol levels.

Kinkade and Wheaton divided fidelity into three components: equipment, environmental, and psychological fidelity[15]. Equipment fidelity refers to the degree to which the equipment of the simulation mimics the equipment of the actual OR, environmental fidelity is the degree to which sensory stimulation is simulated, and psychological fidelity refers to the degree to which skills in the real task are captured by the student. Psychological fidelity depends on both environmental and equipment fidelity. According to Maran and Glavin, it is important that correct cues are given when learning complex tasks to support high-level decision making[16]. Salivary cortisol has a positive correlation with stress and thus can serve as an indirect measure of psychological fidelity[6].

Although the term stress has a negative connotation, it does not necessarily have negative effects. Hans Selye, a Hungarian endocrinologist, first coined the term *eustress* in his 1974 publication *Stress*

*without Distress*. He described eustress as a controlled, manageable stress that does not extend beyond our perceived coping mechanisms[6,17]. Eustress is a positive and motivating force, and is generally of short duration. In 2008, Gibbons et al. found that experiential learning is a source of eustress[18]. Eustress and distress, although perceived differently, both cause similar acute physiological responses, such as increases in heart rate and cortisol levels[19].

Studies of salivary cortisol levels in relation to stress may be extrapolated to evaluate the effectiveness of simulation on learning and memory. In 2009, van Stegeren et al. found that corticosteroid hormones are dose dependent in the enhancement of memory consolidation in humans[20]. Optimal levels of corticosteroid have been shown to enhance poststress declarative memory function[21]. Additionally, Maheu et al. found that inhibition of corticoid synthesis before exposure to stress impairs memory[21].

Simulation provides another avenue for teaching and skill refinement, and is best used as a complement to medical education involving patient care, not as a replacement[22]. Although high-fidelity simulation is generally accepted in most academic settings, more quantitative research is needed in order to determine how simulation is optimally implemented as an educational adjunct[23]. An increase of salivary cortisol levels in students participating in high-fidelity simulation may help to explain the effectiveness of simulation as an educational methodology.

## METHODS

The study was introduced to the incoming class of nurse anesthesia students (n = 68). From this group of students, 21 SRNAs volunteered for the study. All 21 participants provided saliva samples for the baseline data. In the next round of data collection or the simulation experience, 16 of the original 21 participants provided saliva samples for the simulation data. The five missing participants were contacted, but declined to continue in the study due to increased academic demands. In the last round of data collection or the OR clinical experience, 12 of the remaining 16 participants provided salivary samples. The remaining four students were contacted at the clinical sites and declined to continue in the study due to their clinical commitments. Participation was completely voluntary for these students, so there was no repercussion for dropping out of the study. The demographic was then based on the concluding 12 participants' age range from 25 to 37, with a mean age of 29 years. There were 10 females and two males. Participants acted as their own controls throughout the study. The salivary cortisol results were baseline (n = 21), simulation experience (n = 16), and OR clinical experience (n = 12). Any SRNAs who were taking systemic steroids or were unwilling to participate in the study were excluded.

Following the Texas Christian University Institutional Review Board approval**,** participants were provided with Salimetric® Oral Swabs (SOS) and collected baseline saliva samples under nonstimulating conditions, i.e., during the week of spring break. The data collected from these samples served as the control. On the day of simulation, students arrived 10 min prior to their scheduled lab. The simulated patient's history and physical were reviewed. Students verbalized to the panel of three instructors their rationale for medications and type of induction for general anesthesia, either routine induction or rapid-sequence induction. One instructor manipulated the high-fidelity mannequin's voice, cognitive status, and vital signs electronically, and the SRNA was evaluated using standardized criteria according to his or her intervention. After the simulation, the student was informed by one of the instructors of a passing or a nonpassing grade. The clinical collection day varied slightly among participants as some students were involved in direct patient care in the OR, while others simply toured the hospital and completed paperwork.

## RESULTS

The levels of cortisol obtained during simulation were threefold higher than the control cortisol levels (see Fig. 1). Results of the average daily cortisol levels are presented in Fig. 2. Data 1–3 are baseline data for three consecutive mornings. Data 4–6, respectively, represent the morning before simulation lab, the morning of simulation, and the morning after the simulation lab was completed. Data 7–9, respectively, represent the morning before the OR experience, the morning of the OR, and the morning after the OR. Table 1 displays the mean ± the standard error of the mean (SEM). The mean ± SEM of the control data was compared to the mean ± SEM of the laboratory simulation data. The analysis was done using GraphPad Prism 5.0. A two-tailed t test was used to determine differences between the groups. Alpha was set at $p < 0.05$. Control to simulation was threefold higher. There was no difference between control and OR.

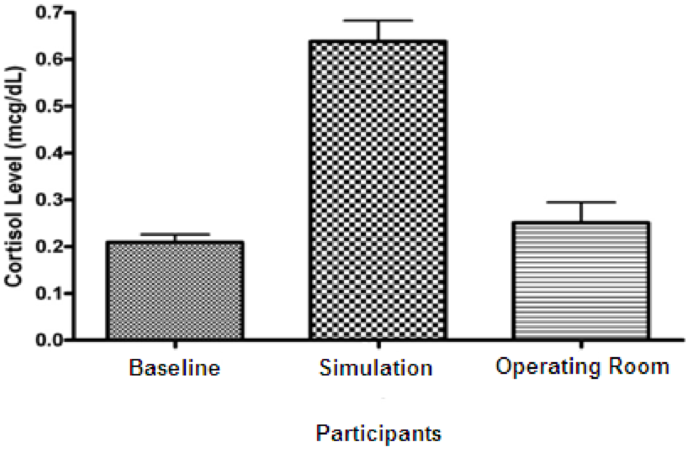

**FIGURE 1**. Mean baseline and simulation cortisol levels.

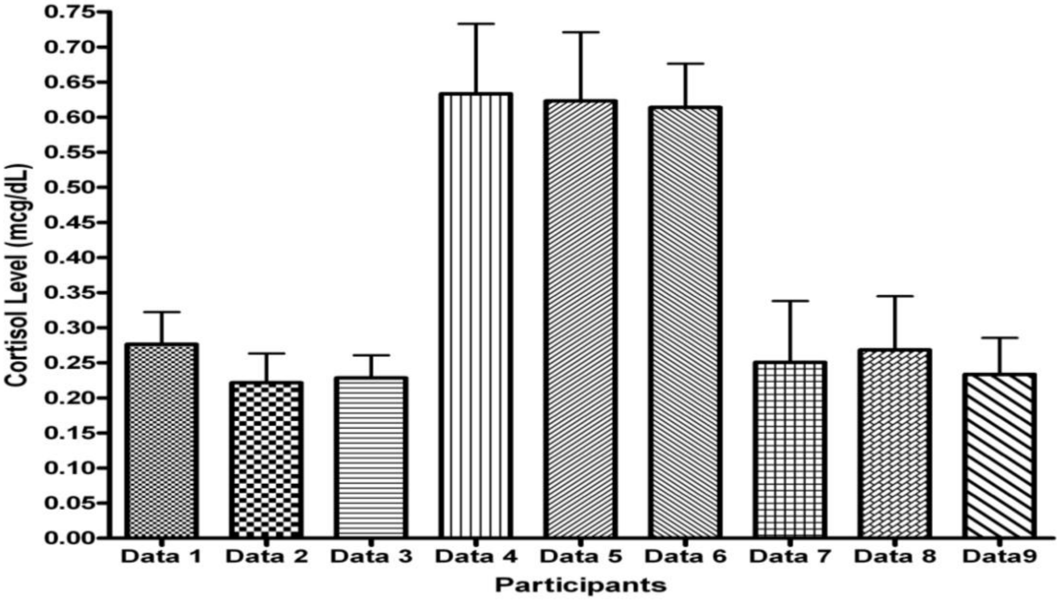

**FIGURE 2.** Average daily cortisol levels in three phases of data collection.

**TABLE 1**
**Results of Study**

| | |
|---|---|
| Control (n = 21) | 0.2088 ± 0.01683 |
| Simulation (n = 16) | 0.7226* ± 0.04370 |
| Operating Room (n = 12) | 0.2508 ± 0.04391 |

* *p* = <0.05 when compared to control.

There were some limitations to the study. Four of 21 students participated in the simulation lab the day before an exam, while six of 21 students attended simulation lab the day of the exam. In both scenarios, cortisol levels were potentially elevated due to the test alone. Such a factor was unavoidable in the design of the study since exam dates could not be altered around the simulation experience. Students were randomly scheduled to complete the simulation lab experience on one of four dates in an attempt to control for this variable as much as possible.

Thus, 10 of 21 students had the potential for increased stress at the time of simulation due to the exam. Conversely, 11 of 21 students performed the simulation experience without an impending exam. After adjusting for the written test, researchers found cortisol levels to be significantly higher than baseline levels among all students (with a *p* value of <0.05).

Another limitation was the inability to control for the first OR day experience or any personal stressors of the SRNAs participating in the study. However, the research team was unaware of any social, psychological, or physical stressors of the subjects during all phases of collection. Lastly, the number of students (n = 12) who completed the study is small relative to the class size (n = 68), and the sample data collection occurred from only one nurse anesthesia program.

## DISCUSSION AND CONCLUSIONS

The purpose of this quasi-experimental study was to explore the cause-effect relationships between independent and dependent variables[24]. Informed consent was obtained by a research assistant to meet the informed consent process for the Institutional Review Board (IRB)[25]. The findings from the study reveal that high-fidelity simulation significantly stressed the participants and increased their cortisol levels above those collected on the first day in the OR. Stress, within a specific range, may actually enhance learning[26]. Although the levels of cortisol during simulation were threefold higher than the control cortisol levels, the cortisol levels remained within normal limits. Therefore, it may be inferred that the stress caused by the simulation lab was eustress, a performance-enhancing stress that engages students[9].

Clinical cortisol levels were comparable to baseline rather than to simulation levels (Figs. 1 and 2, Table 1). One possible explanation for this finding was the administration of an exam within 1 day of the simulation for 10 of the students. The students participating in this study had just completed 12 months of didactic training, so their clinical experience level was equivalent when they entered the OR. In the simulation lab, the students were expected to make independent decisions regarding patient care. In the OR, however, a nurse anesthetist or an anesthesiologist was primarily responsible for the patient and the student took directions for the most part. The first day in the OR is usually quite stressful because of the actual patient involvement, as well as additional professionals and other environmental surroundings not seen in the simulation lab. It is possible that the students' confidence levels and coping mechanisms were improved on the first day of clinical experience as a result of simulation experiences. According to Cannon-Diehl, students who participated in high-fidelity simulation gained confidence and satisfaction with the educational process through the ability to practice complex skills and receive immediate feedback[8]. The implication for practice is that these students will be better acclimated to the stress and activities of the OR during residency due to the realism of simulation.

Simulation appeared to raise cortisol levels above the clinical experience in this study. Future research needs to evaluate learning associated with increased cortisol levels. Perhaps a study of knowledge retention could be compared following a simulation experience with an OR experience. Overall, rigorous studies with fewer limitations over larger populations are needed in order to fully determine the benefit of high-fidelity simulation.
